# miR-26a Reverses Multidrug Resistance in Osteosarcoma by Targeting MCL1

**DOI:** 10.3389/fcell.2021.645381

**Published:** 2021-03-18

**Authors:** Ming Li, Wei Ma

**Affiliations:** Department of Orthopaedic, The Second Hospital of Hebei Medical University, Shijiazhuang, China

**Keywords:** miR-26a, osteosarcoma, chemotherapy, multidrug resistance, MCL1

## Abstract

The multidrug resistance (MDR) acquired in human osteosarcoma is a huge obstacle for effective chemotherapy. Recently, microRNA-26a (miR-26a) has been associated with the pathogenesis and progression of osteosarcoma. However, whether it regulates MDR in osteosarcoma is unknown. We show here that miR-26a expression declines in chemoresistant osteosarcoma after neoadjuvant chemotherapy, and its expression correlates with clinical outcome. In addition, compared with sensitive parental cells, miR-26a expression also declines in osteosarcoma MDR cells, together suggesting a negative correlation between miR-26a expression and MDR development in osteosarcoma. We also show that the enforced expression of miR-26a reverses MDR in osteosarcoma cells, and conversely, miR-26a knockdown confers MDR in chemosensitive osteosarcoma cells treated with doxorubicin, methotrexate, or cisplatin. Mechanistically, miR-26a directly targets the pro-survival protein myeloid cell leukemia 1 (MCL1), and in turn, the enforced expression of MCL1 markedly antagonizes miR-26a-decreased MDR in osteosarcoma MDR cells, therefore demonstrating that miR-26a reverses MDR in osteosarcoma by targeting MCL1. Lastly, miR-26a reverses resistance to doxorubicin in osteosarcoma MDR cells xenografted in nude mice. Collectively, these results reveal a negative role and the underlying mechanism of miR-26a in the regulation of MDR in human osteosarcoma, implying a potential tactic of manipulating miR-26a for overcoming MDR in osteosarcoma chemotherapy.

## Introduction

Osteosarcoma is the most common type of primary bone malignancy in children and adolescents, which remains the leading cause of cancer-related death in adolescents across the world (Luetke et al., 2014; Isakoff et al., 2015; Bray et al., 2018). Owing to surgical resection combined with chemotherapy developed for osteosarcoma, usually consisting of doxorubicin, methotrexate, and cisplatin, the 5-year survival rate of patients with non-metastatic osteosarcoma has been significantly improved to approximately 60–70% over the past decades (Picci, 2007), but for patients with metastatic or recurrent osteosarcoma, the outcome remains poor and the chance of long-term survival is less than 20% despite aggressive therapies (Sakamoto and Iwamoto, 2008). To a large extent, due to the development of multidrug resistance (MDR) either through an intrinsic or acquired manner, chemoresistance becomes the major cause of recurrences and failure of current therapy (Hattinger et al., 2015; Li et al., 2015). It has long been recognized that MDR development in osteosarcoma is mediated by multiple mechanisms, including genetic alterations (Lonning and Knappskog, 2013), altered drug transport (Tiwari et al., 2011), detoxification (Townsend and Tew, 2003), anti-apoptosis, repair of DNA damage, and osteosarcoma stem cells, etc. (Li et al., 2015). However, the mechanisms of MDR development in osteosarcoma are still far from being fully understood.

MicroRNAs (miRNAs) belong to small non-coding regulatory RNA molecules that play vital roles in various processes of tumor biology, and accordingly, their dysregulation has been associated with tumor development, progression, metastasis, survival, apoptosis, and therapy response (Di Leva et al., 2014; Hayes et al., 2014). In recent years, growing evidence has shown that miRNAs regulate MDR in osteosarcoma (Sampson et al., 2015; Liao et al., 2018), such as miR-34b (Zhou et al., 2016), miR-34a-5p (Pu et al., 2017), miR-20a-5p (Zhao et al., 2017), and miR-15b (Duan et al., 2017), to name a few. Previous studies have reported that miR-26a suppresses osteosarcoma tumorigenesis (Wang et al., 2018), migration and invasion (Liu et al., 2018), and tumor growth (Lu et al., 2017). Moreover, its downregulation is associated with metastatic potential and poor prognosis of osteosarcoma patients (Song et al., 2014). These results suggest that miR-26a possesses several anti-osteosarcoma activities, whereas whether it regulates MDR in osteosarcoma and the mechanisms are unclear.

In this study, by investigating clinical osteosarcoma samples and MDR osteosarcoma cell lines, we show that MiR-26a functions to reverse MDR in human osteosarcoma, wherein the targeted expression of myeloid cell leukemia 1 (MCL1) represents a critical mechanism.

## Materials and Methods

### Human Osteosarcoma Specimens

Osteosarcoma patients (*n* = 46) who have undergone chemotherapy and surgical resection of osteosarcoma in the Second Hospital of Hebei Medical University were recruited in this study. Informed consents were obtained from all subjects. This study was approved by the Research Ethics Committee of the Second Hospital of Hebei Medical University. All patients were treated with neoadjuvant chemotherapy consisting of doxorubicin, methotrexate, and cisplatin. The TNM and histological classification of osteosarcoma were performed according to the World Health Organization criteria (Sandberg and Bridge, 2003). After screening, the chemoresistance cohort (*n* = 12, <90% tumor necrosis) and chemosensitive controls (*n* = 9, ≥90% tumor necrosis) after neoadjuvant chemotherapy were identified according to the histologic response described previously (Bacci et al., 2003). All tumor specimens were immediately frozen in liquid nitrogen after resection and stored at −80°C for further analyses. All 46 osteosarcoma patients received a follow-up study. The receiver operating characteristic threshold value of miR-26 expression was used to define the high-expression (*n* = 25) and low-expression groups (*n* = 21).

### Culture and Treatment of Osteosarcoma Cells

Human osteosarcoma cell lines KHOS and U2OS were purchased from ATCC and cultured with complete RPMI 1,640 medium supplemented with 10% fetal bovine serum (FBS) and 100 U/ml penicillin/streptomycin (Thermo Fisher Scientific). The cells were incubated in a humidified atmosphere at 37°C with 5% CO_2_. Their MDR counterparts were generated through the continuous culture of these cells in medium containing step-wise increases in the concentration of paclitaxel for over 8 months as adapted to previous studies (Duan et al., 1999; Yang et al., 2009). Compared to sensitive parental cells, the obtained multidrug-resistant cell lines were resistant to paclitaxel, doxorubicin, mitoxantrone, and vincristine as confirmed by MTT assay as described below.

### IC_50_ Values of Chemotherapeutic Agents in Osteosarcoma Cells

The MDR osteosarcoma cell lines were transfected with mimic NC, miR-26a mimics, antagomir-26a, or antagomir Ctrl 48 h before treatment. The cells were then seeded into the 96-well plates with a density of 1 × 10^4^ cells per well and cultured in a medium containing 10% FBS and a range of doxorubicin, methotrexate, or cisplatin for another 48 h. In detail, to determine the 50% cytotoxic concentration (IC_50_) of chemotherapeutic agents, 10 different concentrations have been used, including 1, 2, 5, 10, 20, 40, 60, 80, 100, and 200 μg/ml. Subsequently, the cells were harvested, and the viable cells were determined using MTT assay (Meng et al., 2017). The IC_50_ value is defined as the concentration of chemotherapeutic agents that results in 50% inhibition of cell proliferation.

### Transfection and Lentiviral Infection-Mediated Stable Overexpression

At 1 day before transfection, KHOS and U2OS cells were plated with a density of 2 × 10^5^ cells per well in six-well plates. For stable transfection, KHOS and U2OS cells were plated at 2 × 10^5^ cells per well in six-well plates. The cells were then transfected with mimic NC, miR-26a mimics, antagomir-26a, or antagomir Ctrl using Lipofectamine 2,000 according to the manufacturer’s instructions. After 48 h of transfection, the cells were used for subsequent analyses. For stable overexpression of miR-26a in KHOS cells, KHOS cells were infected for 24 h with lentiviral-expressing miR-26a or an empty vector (GeneChem). The KHOS cells were cultured for 48 h following infection. The efficiency of transfection and infection was examined by analyses of real-time quantitative PCR or Western blots as described below.

### RNA Extraction and Real-Time Quantitative PCR

Total RNA was extracted from clinical specimens and cultured cells using TRIzol reagent (Thermo Fisher Scientific) according to the manufacturer’s instructions. To measure the expression of miR-26a, real-time quantitative PCR was performed using SYBR Green Master Mix (GenStar) on an ABI 7,500 system (Applied Biosystems) according to the manufacturer’s instructions. Specific primers for amplifying miR-26a and U6 snRNA were synthesized from Ribobio (Guangzhou, China). U6 snRNA was used as an endogenous control. The results were calculated by 2^–Δ^ ^Δ^ ^CT^ method and expressed as a fold change to control (Livak and Schmittgen, 2001).

### Luciferase Reporter Assay

The luciferase reporter assay was conducted as previously described (Chuong et al., 2016). In brief, the 3′-UTR of human MCL1 gene was amplified by PCR and then cloned into the pcDNA3.1 vector (Thermo Fisher Scientific) to construct the recombinant wild-type plasmid named pcDNA3.1-MCL1-wt. The corresponding mutant of 3′-UTR of human MCL1 gene was developed *via* Q5 Site-Directed Mutagenesis Kit (NEB) to construct the recombinant mutant plasmid named pcDNA3.1-MCL1-mut. The sequence of constructs was confirmed by bidirectional sequencing. KHOS cells were seeded in 24-well plates and co-transfected with miR-26a mimics, miR-NC, antagomir-26a, or antagomir Ctrl with luciferase reporter constructs and Renilla luciferase pRL-TK vector (Promega) *via* Lipofectamine 2,000 (Thermo Fisher Scientific) following the manufacturer’s instructions. After 48 h of transfection, the luciferase activity was determined using the Dual-Luciferase Reporter Kit (Promega) based on the manufacturer’s instructions. Finally, the firefly luciferase activity was normalized to the Renilla luciferase activity.

### Western Blots

After treatment, the cells were washed with ice-cold phosphate-buffered saline and collected through centrifugation at 12,000 rpm. The cells were then lysed using RIPA lysis buffer supplemented with protease inhibitor cocktail (Roche). For extracting total proteins from tumor tissues, which were ground into powder with liquid nitrogen prior to lysis using RIPA lysis buffer, the supernatants were collected, and the protein concentration was quantified by bicinchoninic acid assay kit (Beyotime). Aliquots of samples with equal amounts of protein were fractionated by sodium dodecyl sulfate–polyacrylamide gel electrophoresis and then transferred to polyvinylidene difluoride membranes (Millipore). After transferring, the membranes were blocked for 1 h at room temperature with 5% skim milk and then probed overnight with primary antibodies against targets including MCL1 (Cell Signaling, 1:500 dilution) and β-actin (Santa Cruz, 1:2000 dilution). After washing with Tris-buffered saline and Tween 20, the membranes were incubated with corresponding horseradish peroxidase-conjugated secondary antibodies (Santa Cruz) for 1 h at room temperature. The protein blots were detected with ECL Detection System (Thermo Fisher Scientific) and analyzed by ImageJ software.

### *In vivo* Osteosarcoma Xenograft and Treatment

A total number of 5 × 10^5^ MDR KHOS cells overexpressing miR-26a or vector control were resuspended (1:1; v/v) in Matrigel (BD Biosciences) and subcutaneously injected into the right hind flanks of 8-week-old male BALB/c (nu/nu) mice (National Rodent Laboratory Animal Resource, Shanghai). At 1 week after injection, the mice were divided into different groups administrated every 2 days for 3 weeks with or without 5 mg/kg doxorubicin when the tumors reached a volume of 50 mm^3^. Meanwhile, an equivalent volume of sterile saline was injected as a treatment control. The length and width of tumors were measured by a digital caliper starting from treatment day, and the tumor volume was calculated as *W*^2^ × *L*/2. All tumors did not exceed 1,000 mm^3^ until the mice were sacrificed. All animal experiments were conducted in accordance with the protocols approved by the Second Hospital of Hebei Medical University on Research Animal Care.

### Statistical Analysis

The correlation between miR-26a expression and patient prognosis was analyzed by Kaplan–Meier method. The results are expressed as mean ± SD. The significance of difference was calculated using Student’s *t*-test or ANOVA test. Values of *P* < 0.05 were considered statistically significant.

## Results

### miR-26a Declines in Chemoresistant Osteosarcoma and MDR Cells

**FIGURE 1:**
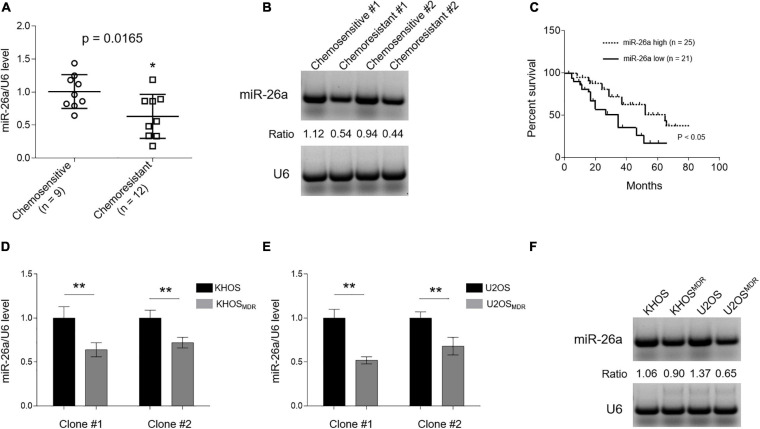
MIR-26a is downregulated in chemoresistant osteosarcoma and multidrug resistance (MDR) cells. **(A)** MiR-26a expression in chemoresistant (*n* = 12) and chemosensitive osteosarcoma (*n* = 9) was determined by real-time quantitative PCR analysis. The expression of U6 was used as an internal control. Each symbol represents the mean value from five replicates. The results are expressed as relative to the chemosensitive group. Data were analyzed using Student’s *t*-test. *P < 0.05. **(B)** Two representative cases from chemoresistant and chemosensitive osteosarcoma samples were analyzed by Northern blotting analysis, and representative images were shown. **(C)** Kaplan–Meier analysis of the overall survival in 46 osteosarcoma patients stratified based on low and high expression of miR-26a. **(D,E)** miR-26a expression in human osteosarcoma cells, including paired sensitive and MDR KHOS **(D)** and U2OS **(E)**, was determined by real-time quantitative PCR analysis. The expression of U6 was used as an internal control. Each column represents the mean value from five replicates. The results are expressed as relative to the parental group. Two obtained individual clones (clone #1 and clone #2) of MDR KHOS and MDR U2OS were examined. **(F)** Clone #1 of MDR KHOS and MDR U2OS cells was analyzed by Northern blotting analysis, and representative images were shown. Data are mean ± SD. Data were analyzed using Student’s *t*-test. **P < 0.01.

Although miR-26a is downregulated in osteosarcoma tissues and cell lines compared with normal controls (Song et al., 2014), whether its expression differs between chemosensitive and chemoresistant cohorts is unclear. To address it, we first compared miR-26a level in clinical osteosarcoma samples from chemoresistant cases (*n* = 12, <90% tumor necrosis) with tumor stage-matched chemosensitive controls (*n* = 9, ≥90% tumor necrosis) after neoadjuvant chemotherapy consisting of doxorubicin, methotrexate, and cisplatin. As revealed by qRT-PCR analysis, miR-26a expression significantly declined in chemoresistant osteosarcoma (Figure 1A), which was further validated by Northern blotting analysis (Figure 1B). Furthermore, the Kaplan–Meier survival analysis showed that patients with high miR-26a expression survived significantly longer than those with low miR-26a expression (Figure 1C) as calculated using the log rank test. This data coincides with a previous study describing that miR-26a loss is an independent prognostic marker for disease-free and overall survival in osteosarcoma (Song et al., 2014). Given miR-26a downregulation in chemoresistant osteosarcoma, we speculated that the correlation of miR-26a with these clinical features might be associated with altered chemotherapy response. Next, we asked whether miR-26a expression declines in human osteosarcoma cells with MDR. Indeed we found that, compared with chemosensitive counterparts KHOS (Figures 1D,F—left half) and U2OS (Figures 1E,F—right half), miR-26a expression was all decreased in those clones of osteosarcoma cells with MDR, which was developed by continuous culture with step-wise increases in the concentration of paclitaxel for over 8 months (Yang et al., 2009). These paclitaxel-resistant cells have indeed upregulated the expression of P-glycoprotein (Supplementary Figure 1), a protein known to play an important role in paclitaxel resistance in cancers, including osteosarcoma (Joshi et al., 2017). Overall, these data establish a negative correlation between miR-26a expression and MDR in human osteosarcoma, hinting a possible functional role of miR-26a in osteosarcoma chemoresistance.

### Enforced miR-26a Expression Reverses MDR in Osteosarcoma Cells

**FIGURE 2:**
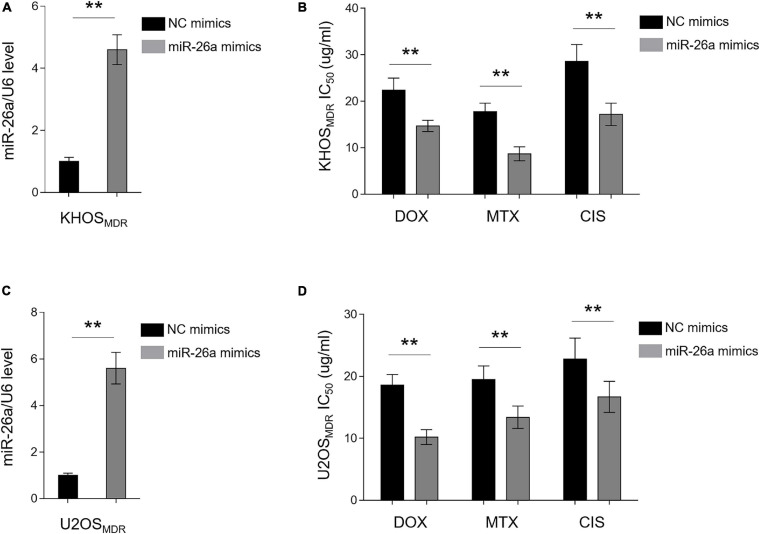
miR-26a reverses multidrug resistance (MDR) in osteosarcoma MDR cells. **(A,B)** MDR KHOS cells were transfected with miR-26a mimic or mimic control for 48 h. **(A)** miR-26a expression was determined by qRT-PCR analysis. The expression of U6 was used as an internal control. Each column represents the mean value from five replicates. The results are expressed as relative to the control group. Data were analyzed using Student’s *t*-test. **P < 0.01. **(B)** Cells were then treated with doxorubicin, methotrexate, or cisplatin for another 48 h. The 50% cytotoxic concentration (IC_50_) was evaluated by MTT assay. Each column represents the mean value from five replicates. Data were analyzed using Student’s *t*-test. **P < 0.01. **(C,D)** MDR U2OS cells were treated as in **(A)**. miR-26a expression **(C)** and IC_50_ value **(D)** were evaluated and presented as in **(A,B)**.

The high expression of miR-26a has been shown to be associated with favorable outcome on tamoxifen in metastatic breast cancer (Jansen et al., 2012). In addition, decreased miR-26a expression was found to cause cisplatin resistance in human non-small cell lung cancer (Yang et al., 2016). Together with our data above showing that miR-26a is downregulated in chemoresistant human osteosarcoma (Figure 1), we speculated that miR-26a is very likely to affect MDR in osteosarcoma. To demonstrate it, we performed an enforced expression of miR-26a in MDR osteosarcoma cells through the transient transfection of miR-26a mimics. The enforced expression of miR-26a in MDR KHOS cells was confirmed by qRT-PCR analysis (Figure 2A). MDR KHOS cells were then treated with increasing concentrations of doxorubicin, methotrexate, or cisplatin, and the IC_50_ value was evaluated by MTT assay. As shown in Figure 2B, MDR KHOS cells with enforced miR-26a expression had lower IC_50_ value for all three chemotherapeutic drugs compared with those transfected with NC mimics. This observation was reproduced by the CCK-8 assay (Supplementary Figure 2). Moreover, similar results were obtained when MDR U2OS were analyzed (Figures 2C,D). These results together indicate that the enforced expression of miR-26a reduces MDR in osteosarcoma cell lines and resensitizes them to the treatment of chemotherapeutic drugs.

### miR-26a Knockdown Confers MDR in Chemosensitive Osteosarcoma Cells

**FIGURE 3:**
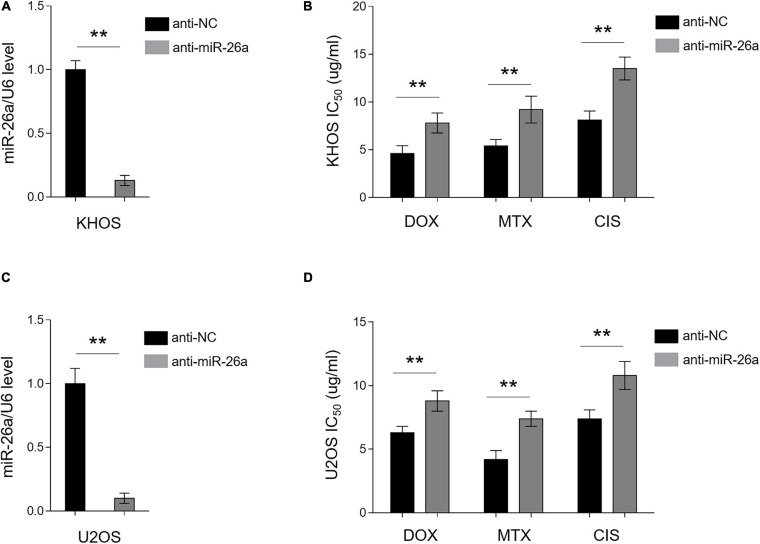
miR-26a knockdown confers multidrug resistance in chemosensitive osteosarcoma cells. **(A,B)** KHOS cells were transfected with anti-miR-26a or anti-NC for 48 h. **(A)** miR-26a expression was determined by qRT-PCR analysis. The expression of U6 was used as an internal control. Each column represents the mean value from five replicates. The results are expressed as relative to the control group. Data were analyzed using Student’s *t*-test. **P < 0.01. **(B)** Cells were then treated with doxorubicin, methotrexate, or cisplatin for another 48 h. The 50% cytotoxic concentration (IC_50_) was evaluated by MTT assay. Each column represents the mean value from five replicates. Data were analyzed using Student’s *t*-test. **P < 0.01. **(C,D)** U2OS cells were treated as in **(A)**. miR-26a expression **(C)** and IC_50_ value **(D)** were evaluated and presented as in **(A,B)**.

In an opposite direction, we further confirmed the role of miR-26a in osteosarcoma MDR by performing knockdown assay *via* transfecting anti-NC or anti-miR-26a into chemosensitive human osteosarcoma cells KHOS and U2OS. The knockdown efficiency of miR-26a in KHOS (Figure 3A) and U2OS (Figure 3C) was verified by qRT-PCR analysis. Functionally, in agreement with results from miR-26a enforced expression (Figure 2), miR-26a knockdown conversely resulted in markedly higher IC_50_ value in KHOS (Figure 3B) and U2OS (Figure 3D) treated with all three chemotherapeutic drugs as compared with those transfected with anti-NC. These data illustrate that miR-26a knockdown confers MDR in osteosarcoma cells and renders them more resistant to the treatment of chemotherapeutic drugs. Together with Figure 2, these lines of evidence prove that miR-26a functions as a negative regulator of MDR in human osteosarcoma.

### miR-26a Directly Targets MCL1 in Osteosarcoma Cells

**FIGURE 4:**
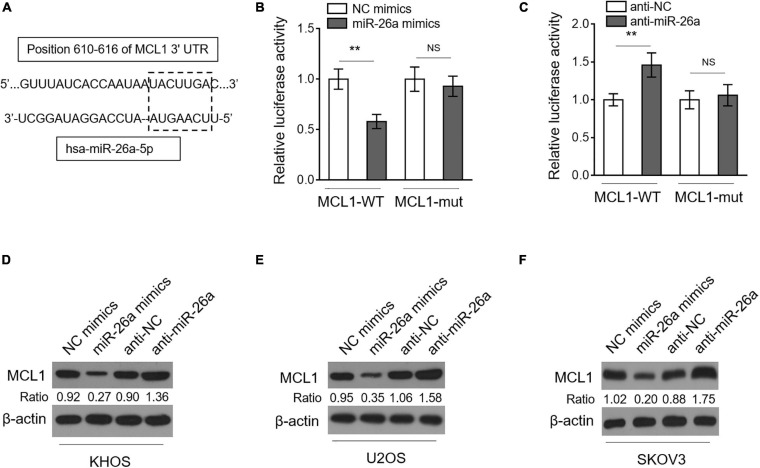
miR-26a directly targets MCL1 in osteosarcoma cells. **(A)** Schematic illustration of the complementary sequence (within the gridline) between miR-26a and the 3’-UTRs of MCL1 mRNA. This information is provided by the computational and bioinformatics-based approach using TargetScan. **(B)** KHOS cells were co-transfected with pMIR-LUC-3’-UTR-MCL1-wt (MCL1-wt) or MCL1-mut with miR-26a mimics or NC mimics. Following 48 h of transfection, luciferase activity was measured. Each column represents the mean value from five replicates. The results relative to the control group are shown. Data were analyzed using Student’s *t*-test. **P < 0.01. NS, not significant. **(C)** KHOS cells were co-transfected with pMIR-LUC-3’-UTR- MCL1-wt (MCL1-wt) or MCL1-mut with anti-miR-26a or anti-NC. Following 48 h of transfection, luciferase activity was measured. Each column represents the mean value from five replicates. The results relative to the control group are shown. Data were analyzed using Student’s *t*-test. **P < 0.01. NS, not significant. KHOS **(D)**, U2OS **(E)**, and SKOV3 **(F)** cells were transfected with miR-26a mimics or NC mimics or with anti-miR-26a or anti-NC as indicated for 48 h. The expression of MCL1 was determined by Western blotting analysis. β-Actin was used as a loading control. Three independent experiments were conducted, and the representative images are shown.

To elucidate how miR-26a regulates MDR in osteosarcoma, we predicted targets of miR-26a through open-access databases including TargetScan ver. 7.2 and miRanda ver. 1.9. Among the potential candidates (Supplementary Table 1), MCL1 (Figure 4A), a protein belonging to the Bcl-2 family that plays critical anti-apoptotic roles, attracted our attention since it confers MDR in acute myeloid leukemia (Hermanson et al., 2013) and is also positively related to drug resistance in several types of cancer (Campbell et al., 2010; Sugio et al., 2014; Ou et al., 2015; Badejo et al., 2016). We next conducted dual-luciferase reporter analysis to verify whether miR-26a directly targets the 3′-UTR of MCL1 in human osteosarcoma cells. The results showed that the co-expression of miR-26a significantly suppressed the luciferase activity of the construct containing a wild-type 3’-UTR of MCL1 but did not suppress that containing a mutant 3’-UTR of MCL1 in KHOS cells (Figure 4B). The direct binding of MCL1 to miR-26a was further confirmed by the reverse proof that anti-miR-26a transfection-induced miR-26a downregulation significantly increased the luciferase activity of the construct containing a wild-type 3’-UTR of MCL1 but did not increase that containing a mutant 3’-UTR of MCL1 in KHOS cells (Figure 4C). As a target of miR-26a in human osteosarcoma cells, the expression of MCL1 ought to be suppressed by miR-26a. Indeed we found that KHOS cells transfected with miR-26a mimics had a lower MCL1 expression, and conversely, those transfected with anti-miR-26a resulted in a higher MCL1 expression (Figure 4D). Moreover, similar results were obtained when analyzing U2OS (Figure 4E) and SKOV3 (Figure 4F). Taken together, these results identify MCL1 as a direct target of miR-26a in human osteosarcoma cells, whereby miR-26a decreases MCL1 expression.

### MCL1 Restoration Diminishes miR-26a-Reversed MDR in Osteosarcoma MDR Cells

**FIGURE 5:**
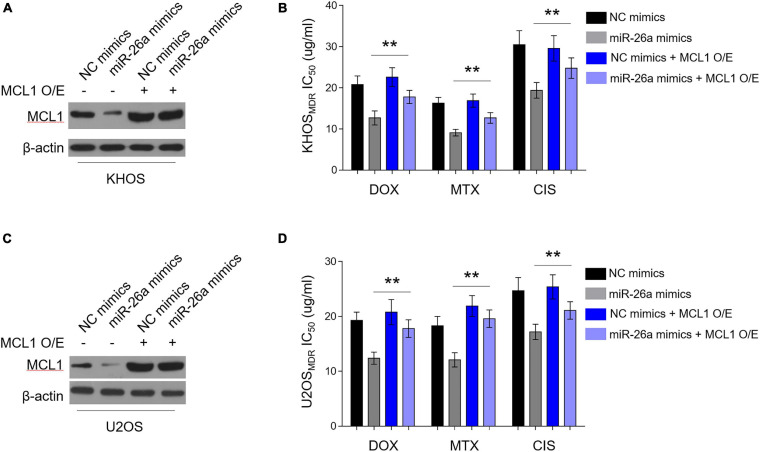
Enforced MCL1 expression restores miR-26a-reversed multidrug resistance (MDR) in osteosarcoma MDR cells. **(A,B)** MDR KHOS cells stably infected with lentivirus-expressing vector or MCL1 were transfected with miR-26a mimics or NC mimics for 48 h. O/E, overexpression. **(A)** MCL1 expression was determined by Western blotting analysis. β-Actin was used as a loading control. Three independent experiments were conducted, and the representative images are shown. **(B)** Cells were then treated with doxorubicin, methotrexate, or cisplatin for another 48 h. The 50% cytotoxic concentration (IC_50_) was evaluated by MTT assay. Each column represents the mean value from five replicates. Data were analyzed using Student’s *t*-test. **P < 0.01. **(C,D)** MDR U2OS cells were treated as in **(A)**. MCL1 expression **(C)** and IC_50_ value **(D)** were evaluated and analyzed as in **(A,B)**.

We asked whether miR-26a-decreased MCL1 expression contributes to the reversed MDR in human osteosarcoma cells. For this purpose, we overexpressed MCL1 in osteosarcoma MDR cells, which were simultaneously enforced to express miR-26a. Consistently, the enforced expression of miR-26a *via* transfection of miR-26a mimics resulted in a reduction of MCL1 expression in KHOS cells, and this reduction of MCL1 expression by miR-26a was reversed in the presence of MCL1 overexpression (Figure 5A), demonstrating the completely restored expression of MCL1 mediated by overexpression. Importantly, along with the restored MCL1 expression, the lower IC_50_ value for all three chemotherapeutic drugs caused by miR-26a enforcement was substantially recovered (Figure 5B). Furthermore, similar results were obtained in U2OS (Figures 5C,D). These findings indicate that MCL1 largely restores miR-26a-reversed MDR in human osteosarcoma cells to resensitize these cells to the treatment of chemotherapeutic drugs. Thus, a critical mechanism by which miR-26a negatively regulates MDR in human osteosarcoma is through targeting and suppressing MCL1 expression.

### MiR-26a Reverses Doxorubicin Resistance in Osteosarcoma MDR Cells *in vivo*

**FIGURE 6:**
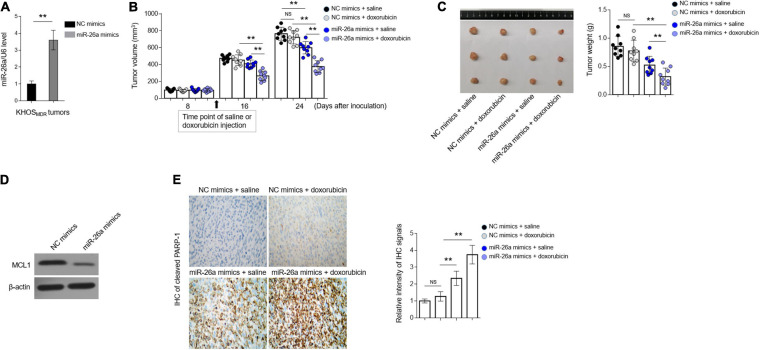
miR-26a reverses doxorubicin resistance in osteosarcoma multidrug resistance (MDR) cells *in vivo*. **(A)** MDR KHOS cells were transfected with miR-26a mimic or mimic control for 48 h *in vitro* and then xenografted into nude mice (*n* = 9). The mice were intraperitoneally injected with chemotherapeutic drugs every 2 days for 3 weeks. miR-26a expression in tumors was determined by qRT-PCR analysis. The expression of U6 was used as an internal control. Each column represents the mean value from five replicates. The results are expressed as relative to the control group. Data were analyzed using Student’s *t*-test. **P < 0.01. **(B)** The tumor volume of MDR KHOS cells treated with doxorubicin was calculated and monitored at 8, 16, and 24 days following injection. **(C)** The representative images of resected tumor (left half) and tumor weight (right half) are shown. Each symbol represents the value of each tumor. **(D)** The protein expression of MCL1 in the resected tumors was determined by Western blotting analysis. The representative images are shown. **(E)** The expression of cleaved PARP-1 was detected in the slices of isolated tumors by immunohistochemistry (IHC) analysis. The intensity of the IHC signals is shown on the right. Data are mean ± SD. Data were analyzed using one-way ANOVA. **P < 0.01. NS, not significant.

To broaden our findings into *in vivo* settings, we lastly tested whether miR-26a regulates MDR in human osteosarcoma xenografted in immunodeficient nude mice. KHOS cells stably overexpressing miR-26a *via* lentiviral infection were inoculated into the right hind flanks of BALB/c (nu/nu) mice, followed by intraperitoneal administration every 2 days for 3 weeks with 5 mg/kg doxorubicin, and during this process, the volume of tumor was measured, and the growth curve was depicted. In the end, the expression of miR-26a in inoculated tumors was measured, and the lentiviral infection-mediated overexpression of miR-26a was confirmed by qRT-PCR analysis (Figure 6A). More importantly, as shown in Figures 6B,C, compared with the treatment of vehicle saline, doxorubicin treatment alone had no obvious effects on suppressing tumor growth, illustrating the MDR feature of KHOS cells maintained *in vivo*. miR-26a overexpression alone exhibited a suppressive effect on tumor growth when treated with vehicle saline (*P* < 0.02) at 24 days after inoculation (Figures 6B,C), which is consistent with a previous report demonstrating that miR-26a possesses anti-growth activity in xenografted osteosarcoma model (Lu et al., 2017). Furthermore, when treated with doxorubicin in combination, compared with those expressing NC, miR-26a-overexpressing KHOS cells developed significantly smaller tumors (*P* < 0.001), even much smaller than that of miR-26a-overexpressing KHOS treated with vehicle saline (*P* < 0.001) (Figures 6B,C). In line with these results, miR-26a overexpression led to reduced expression of anti-apoptotic protein MCL1 (Figure 6D) and significant induction of tumor cell apoptosis, as evidenced by elevated expression of cleaved PARP-1, which was further enhanced when doxorubicin was treated in combination (Figure 6E). These observations indicate not only that the profound suppressive effect on growth of miR-26a-overexpressing KHOS cells treated with doxorubicin is not derived from the anti-growth activity of miR-26a itself but also that this effect is caused by resensitization to doxorubicin treatment.

In conclusion, together with results from clinical samples and several *in vitro* evidence, this study identifies miR-26a as a novel regulator of MDR in human osteosarcoma cells, in which the targeted expression of MCL1 is an important mechanism. We therefore may provide a molecular basis for the possible application of miR-26a in overcoming MDR developed in osteosarcoma.

## Discussion

For almost the last two decades, high-dose and multi-drug neoadjuvant chemotherapy, primarily consisting of adriamycin, cisplatin, methotrexate, ifosfamide, and epirubicin, has been used as the gold-standard treatment for patients with osteosarcoma (Su et al., 2015). Although neoadjuvant chemotherapy significantly improves prognosis, due to drug resistance and/or intensive side effects, many patients are often forced to terminate or modify chemotherapy, which results in deteriorated disease progression and poor clinical outcome (Harrison et al., 2018). Therefore, novel agents and strategies are needed to combat chemoresistant osteosarcoma. Concerning chemoresistance, accumulating evidence has shown that miRNAs play a vital role in MDR through regulating various drug-resistant mechanisms, i.e., defects in the apoptotic machinery (An et al., 2017), thus holding promise for the development of more effective therapies for MDR osteosarcoma. This study was designed to investigate whether miR-26a, recently found to predict the prognosis of osteosarcoma patients (Song et al., 2014), is associated with and plays a functional role in MDR in osteosarcoma.

As shown by the evidence, we report, for the first time, the decreased expression of miR-26a in chemoresistant osteosarcoma sampled from patients treated with neoadjuvant chemotherapy consisting of doxorubicin, methotrexate, and cisplatin. In addition, miR-26a expression is also decreased in MDR osteosarcoma cell lines established by continuous selection with the mixture of doxorubicin, methotrexate, and cisplatin. Evidence from these clinical and *in vitro* models, showing that miR-26a expression is decreased in chemoresistant osteosarcoma compared with chemosensitive counterparts, implies that miR-26a may be involved in the regulation of MDR in osteosarcoma. Indeed our subsequent functional studies *via* enforced and knockdown of miR-26a expression indicate that miR-26a plays a negative role in MDR in human osteosarcoma in both *in vitro* and *in vivo* settings. Furthermore, by predicting the targets of miR-26a, we reveal that miR-26a directly targets and decreases MCL1 expression in osteosarcoma cells. Lastly, the reverse proof that enforced MCL1 expression markedly restores miR-26a-reversed MDR in osteosarcoma MDR cell lines demonstrates that the targeted expression of MCL1 is a critical mechanism by which miR-26a negatively regulates MDR in osteosarcoma. In sum, given the role and mechanism underlying miR-26a regulation of MDR in osteosarcoma, our study may provide molecular basis for the application of targeting miR-26a in osteosarcoma to reverse MDR and improve the effectiveness of chemotherapy.

Consistent with a previous study (Song et al., 2014), we also found that miR-26a expression is negatively correlated with a poor prognosis of osteosarcoma patients. Nonetheless, one caveat is that the size of the recruited clinical samples is small to better relate the expression change of miR-26a to osteosarcoma chemoresistance. Further investigations with a larger sample size are warranted to consolidate our conclusion that miR-26a is decreased in chemoresistant osteosarcoma. Moreover, whether miR-26a expression displays a similar change in chemoresistant osteosarcoma treated with other chemotherapy regimens is also uncertain. This issue is also applied to miR-26a expression in MDR osteosarcoma cell lines used in our study since they were selected by the same chemotherapeutic agents. Addressing this issue may help to confirm whether miR-26a is associated with the chemoresistance of osteosarcoma across a wide spectrum.

Of note is the observation that the restoration of MCL1 largely rescues the miR-26a-reversed MDR in osteosarcoma, suggesting that there exist other mechanisms that may be responsible for miR-26a regulation in MDR in osteosarcoma. To date, except for MCL1 which has been proven by us and other literature studying hepatocellular carcinoma (Gao et al., 2018), among the validated miR-26a targets, HMGA1 (Liu et al., 2018), PTEN (Liu et al., 2012), EZH2 (Lu et al., 2011), MITF (Ji et al., 2015), DNMT3B (Lai et al., 2019), TGF-beta (Tripathi et al., 2019), and Ezh2 (Gollner et al., 2017) have been demonstrated to regulate chemoresistance. For instance, HMGA1 is able to regulate chemoresistance in ovarian cancer stem cells (Kim et al., 2016) and non-small cell lung cancer cells (Wang et al., 2017). Therefore, it is of interest to examine whether these candidates also play a role in mediating miR-26a function in MDR in osteosarcoma. MCL1 is well established as an anti-apoptotic molecule which could regulate chemoresistance in multiple cancer types, including osteosarcoma (Osaki et al., 2014). On the other hand, as known, the development of MDR in osteosarcoma is a highly complicated process in which a variety of mechanisms are involved (An et al., 2017), and conceivably, due to the extensive range of targets, miR-26a probably regulates MDR in osteosarcoma through other mechanisms. Deeper studies are needed to discover the detailed mechanisms through which miR-26a regulates MDR in osteosarcoma.

In summary, in addition to its suppressive effect on proliferation, growth, stem cell-like property, and invasion of osteosarcoma, our study identifies miR-26a as a negative regulator of MDR in osteosarcoma, hence providing another anti-osteosarcoma activity by which miR-26a influences osteosarcoma progression and therapy response.

## Data Availability Statement

The raw data supporting the conclusions of this article will be made available by the authors, without undue reservation.

## Ethics Statement

The studies involving human participants were reviewed and approved by the Second Hospital of Hebei Medical University. The patients/participants provided their written informed consent to participate in this study. The animal study was reviewed and approved by the Second Hospital of Hebei Medical University.

## Author Contributions

WM conceived and designed the current study and contributed to writing the manuscript. ML performed the experiments, analyzed, and interpreted the data. Both authors read and approved the final manuscript.

## Conflict of Interest

The authors declare that the research was conducted in the absence of any commercial or financial relationships that could be construed as a potential conflict of interest.

## Supplementary Material

Supplementary Figure 1 P-glycoprotein expression in paired sensitive and multidrug resistance KHOS and U2OS was determined by Western blotting analysis. The expression of β-actin was used as a loading control. The representative images were shown. Data are mean ± SD. Data were analyzed using Student’s *t*-test. ^∗∗^*P* < 0.01.

Supplementary Figure 2 Cells were then treated with doxorubicin, methotrexate, or cisplatin for another 48 h. The 50% cytotoxic concentration was evaluated by CCK-8 assay. Each column represents the mean value from five replicates. Data were analyzed using Student’s *t*-test. ^∗∗^*P* < 0.01.
