# Supplementary figures and images for: miR-26a Reverses Multidrug Resistance in Osteosarcoma by Targeting MCL1
Source: Front Cell Dev Biol. 2021 Mar 18;9:645381. doi: 10.3389/fcell.2021.645381 (PMC8012539; doi:10.3389/fcell.2021.645381)

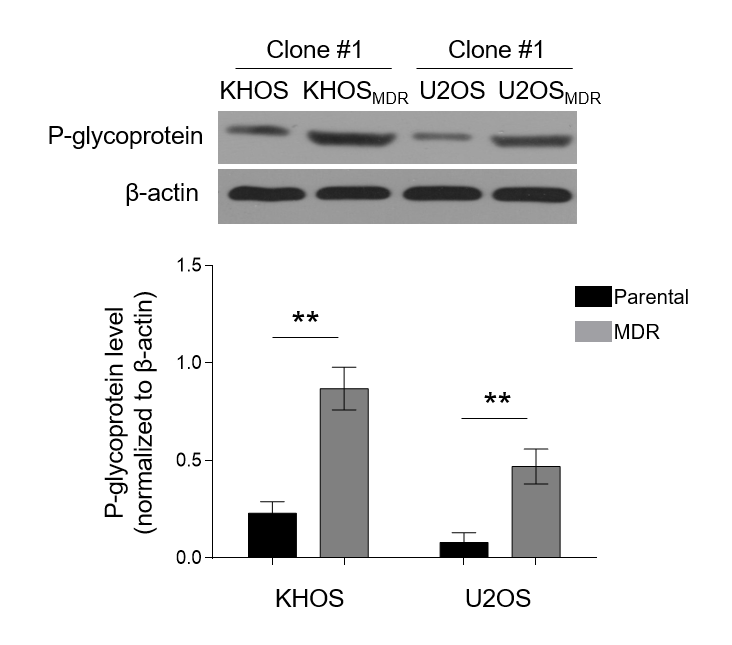

Supplement: Supplementary Figure 1 — P-glycoprotein expression in paired sensitive and multidrug resistance KHOS and U2OS was determined by Western blotting analysis. The expression of β-actin was used as a loading control. The representative images were shown. Data are mean ± SD. Data were analyzed using Student’s t-test. ∗∗P < 0.01. [file Image_1.TIF]

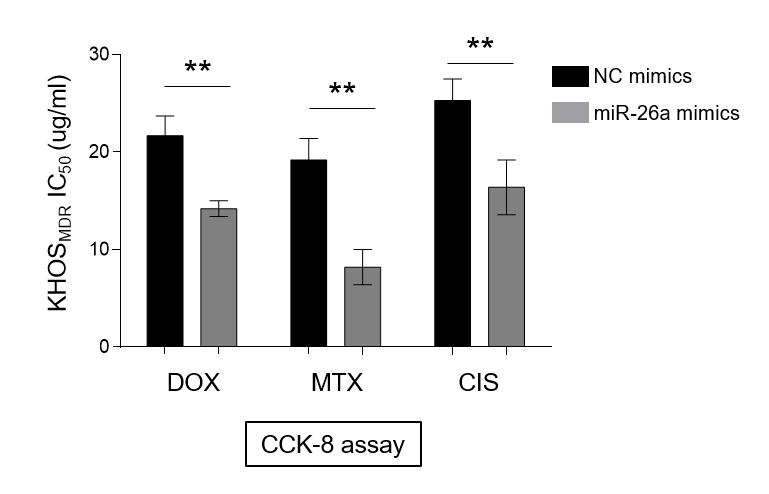

Supplement: Supplementary Figure 2 — Cells were then treated with doxorubicin, methotrexate, or cisplatin for another 48 h. The 50% cytotoxic concentration was evaluated by CCK-8 assay. Each column represents the mean value from five replicates. Data were analyzed using Student’s t-test. ∗∗P < 0.01. [file Image_2.TIF]
